# A bioavailable form of curcumin, in combination with vitamin-D- and omega-3-enriched diet, modifies disease onset and outcomes in a murine model of collagen-induced arthritis

**DOI:** 10.1186/s13075-021-02423-z

**Published:** 2021-01-25

**Authors:** Mahadevappa Hemshekhar, Vidyanand Anaparti, Hani El-Gabalawy, Neeloffer Mookherjee

**Affiliations:** 1 Manitoba Centre for Proteomics and Systems Biology, Department of Internal Medicine, University of Manitoba, 799 John Buhler Research Centre, 715 McDermot Ave, Winnipeg, MB Canada; 2 Division of Rheumatology, Department of Internal Medicine, University of Manitoba, Winnipeg, MB Canada; 3 Department of Immunology, University of Manitoba, Winnipeg, MB R3E3P4 Canada

**Keywords:** Rheumatoid arthritis, Inflammation, Curcumin, Vitamin D, Omega-3, Collagen-induced arthritis

## Abstract

**Objective:**

Curcumin (CUR), vitamin D_3_ (D3), and omega-3-fatty acids (O3FA) individually modulate inflammation and pain in arthritis. Although these supplements are widely used, their combinatorial effects have not been defined. In this study, we examined the effects of a D3 and O3FA (VO)-enriched diet in conjunction with a highly bioavailable form of CUR (Cureit/Acumin™) in a collagen-induced arthritis (CIA) murine model.

**Methods:**

Male DBA/1J mice were acclimatized to VO-enriched diet and challenged with bovine collagen II (CII). Bioavailable CUR was administered daily by oral gavage from the onset of CII challenge. Disease severity was determined by monitoring joint thickness and standardized clinical score. Cellular infiltration and cartilage degradation in the joints were assessed by histology, serum cytokines profiled by Meso Scale Discovery multiplex assay, and joint matrix metalloproteinases examined by western blots.

**Results:**

CUR by itself significantly decreased disease severity by ~ 60%. Administration of CUR in CIA mice taking a VO-enriched diet decreased disease severity by > 80% and maximally delayed disease onset and progression. Some of the disease-modifying effects was mediated by CUR alone, e.g., suppression of serum anti-collagen antibodies and decrease of cellular infiltration and MMP abundance in the joints of CIA mice. Although CUR alone suppressed inflammatory cytokines in serum of CIA mice, the combination of CUR and VO diet significantly enhanced the suppression (> 2-fold compared to CUR) of TNF, IFN-γ, and MCP-1, all known to be associated with RA pathogenesis.

**Conclusion:**

This study provides proof-of-concept that the combination of bioavailable CUR, vitamin D_3_, and O3FA substantially delays the development and severity of CIA. These findings provide a rationale for systematically evaluating these widely available supplements in individuals at risk for developing future RA.

**Supplementary Information:**

The online version contains supplementary material available at 10.1186/s13075-021-02423-z.

## Introduction

Rheumatoid arthritis (RA) is a systemic autoimmune disease that affects nearly 1% of the adult population worldwide [1, 2]. It is characterized by immune-mediated chronic inflammation targeting the synovium of multiple joints which, if not treated early, results in irreversible degradation of the adjacent cartilage and bone leading to progressive deformity and functional loss [1–3]. Treatment with the currently available arsenal of disease-modifying anti-rheumatic drugs (DMARDs), biologics, and Janus-Kinase (JNK) inhibitors, often in combination, has dramatically improved the outcomes of this disease [4, 5]. However, these treatments are associated with significant immunosuppression, prohibitive cost, and in some cases treatment failure to multiple agents. Furthermore, most patients require ongoing therapy to prevent recurrence of the inflammatory process.

RA is known to be associated with specific autoantibodies, in particular the anti-citrullinated protein antibodies (ACPA) [1–3, 6]. Studies in multiple populations have clearly shown that ACPA and other RA-associated autoantibodies are detectable months to years prior to the onset of clinically detectable articular and systemic inflammation [6, 7]. Although the pathogenic role of these autoantibodies continues to be debated, the detection of preclinical ACPA seropositivity has led to the hypothesis that interventions during this stage may serve to substantially reduce the severity of the disease and delay or even prevent disease onset [6, 7]. Although several strategies are currently being evaluated in this context, the safety and acceptability of any intervention at the preclinical stage is a key consideration [6, 7].

Recent studies have demonstrated beneficial effects of specific nutritional supplements in RA [8, 9], which includes curcumin (CUR), vitamin D_3_ (D3), and omega-3-fatty acids (O3FA) [10–14]. This is corroborated with studies demonstrating that decreased serum levels of D3 and O3FA are associated with increased disease activity in RA patients [15, 16]. D3 insufficiency is known to promote inflammation and autoimmune responses [13, 17, 18]. Both D3 and O3FA are known to exert immunomodulatory effects. D3 modulates innate immune responses to promote acute response to infections by engaging macrophages to induce Toll-like receptor target genes and antimicrobial peptides [19]. D3 also mediates anti-inflammatory mechanisms by modulating critical pathways such as p38 MAPK and NF-κB to suppress pro-inflammatory cytokines and prostaglandin [20]. Similarly, O3FA influences cytokine secretion and polarization of macrophages, counteracting inflammatory gene and protein expression by modulating the NF-κB pathway [21]. O3FA also blunts the polarization of Th-17 cells while enhancing the accumulation of Tregs, to skew immune responses towards an anti-inflammatory phenotype [21]. Aligned with these mechanistic studies, independent clinical studies of supplementation with either D3 or O3FA have demonstrated that these supplements can suppress the levels of circulating inflammatory mediators and modulate disease activity in RA [13, 22]. Interestingly, a prospective study showed that increased dietary intake of both D3 and O3FA a year before DMARD initiation enhances treatment benefits, indicating a combinatorial effect of D3 and O3FA in patients with early RA [23]. However, specific molecular determinants that are altered in response to a combination of D3 and O3FA have not yet been defined. Nevertheless, previous studies have demonstrated that the combination of D3 and O3FA mediates anti-inflammatory effects even in concentrations where individually these nutrients have minor effects, likely due to the convergence of signaling pathways to enhance anti-inflammatory responses [24]. Therefore, in this study, we chose to examine the effects of a combinatorial diet of D3 and O3FA, with and without CUR administration. Although CUR has been shown to exert anti-inflammatory effects in arthritis [10, 25, 26], a major challenge associated with its supplementation has been bioavailability [27, 28]. There are no studies to date that have examined the effect of CUR to modify either disease onset or severity, along with D3 and O3FA, in vivo in the context of RA.

There are several animal models of inflammatory arthritis that mimic various aspects and stages of the disease, but no single model recapitulates the entire biological process in RA. In particular, it has proven difficult to model the specific autoimmune processes that underpin seropositive RA. Arguably, the collagen-induced arthritis (CIA) model is proven to be the most informative in modeling the immune-mediated events that precede and precipitate the joint inflammation and destruction [29–31]. Therefore, in this study, we comprehensively examined the effects of a diet enriched in vitamin D_3_ and O3FA (VO-enriched diet) in a CIA model, with and without oral administration of a highly bioavailable form of CUR [32, 33].

## Material and methods

### Collagen-induced arthritis (CIA) murine model

The protocol used for the CIA murine model was based on our previous study [30] and approved by The University of Manitoba Animal Research Ethics Board. Experimental design and reporting of data is compliant with the ARRIVE guidelines for in vivo animal research [34]. DBA/1J male mice (∼ 6 weeks old) were obtained from Jackson laboratories. Subsequently, the animals were divided into two groups: one group fed with a standard diet (a fixed formulation diet for laboratory mice fortified with vitamins and minerals), and the second group was fed a vitamin D_3_ (10,000 IU/kg of diet) and O3FA (10 g/kg of diet)-enriched diet (VO-enriched diet). These diet feeds were obtained from Research diet Inc., New Brunswick, USA. Each mouse was ~ 25 g body weight, and the amount of feed ingested per mouse was estimated to be ~ 5 g per day. Thus, the intake of D3 and O3FA in the VO-enriched diet was estimated to be ~ 50 IU of D3 and ~ 50 mg of O3FA, per day per mouse. Mice were acclimatized for at least 2 weeks for housing environment and the respective diets, before collagen challenge.

Mice in each diet group were further divided into 4 subgroups: (i) unchallenged mice with saline administration as control, (ii) with CUR administration, (iii) CIA-challenged mice, and (iv) CIA-challenged mice with CUR administration. The highly bioavailable form of CUR (Cureit™) was obtained from Aurea Biolabs Ltd. (Cochin, Kerala, India) and administered daily by oral gavage, at a dose of 100 mg/kg per mouse, starting from the same day mice were introduced to standard or VO-enriched diet. The dose of each of the dietary supplement used in this study was based on previous studies in either murine preclinical models and/or extrapolation of use by humans [35–38]. Although previous studies have used CUR at a range of 100–1000 mg/kg body weight of mice, we used a lower dose of CUR considering the high bioavailability of Cureit/Acumin formulation and based on previous studies with this CUR formulation [39–43]. Thus, each of the nutritional supplements used was at a dose range applicable for human use.

Two independent experimental trials were performed with five mice per group per trial, to obtain a cumulative data of *n* = 10 mice per group. The CIA-challenged protocol was based on our previous studies [29, 30]. Briefly, mice were anesthetized using isoflurane and challenged with a subcutaneous (s.c.) tail injection of 100 μg bovine collagen type II (CII) emulsified in complete Freund’s adjuvant in a total volume of 100 μl. A boost of 50 μg CII emulsified in incomplete adjuvant (total volume of 50 μl) was administered (s.c) in the tail on day 21 after the initial CII challenge. Mice were administered with LPS from *E. coli* 0111:B4 (20 μg per mouse) intraperitoneally (i.p.) on day 25 after the first CII challenge [30]. All reagents for the CIA challenge were obtained from Chondrex Inc. (Redmond, WA, USA). Saline (100 μl) was administered (s.c.) in the control mice on day 1 and day 21 after the initial CII challenge, administered orally parallel to CUR administration, and used as control mice. CIA challenge and CUR administration was performed between 10 am and 1 pm on specified days. Mice were anesthetized with isoflurane and euthanized by cardiac puncture on day 29 after the first CII challenge. Blood obtained by cardiac puncture was used to obtain serum, which was aliquoted and stored at − 20 °C until use. Mouse joints were collected, cleaned to remove skin/tissues, processed for histology, and used to obtain protein lysates for analyses.

### Evaluation of disease progression

Mice were visually monitored for grooming and activity levels daily. Disease progression was assessed by monitoring joint thickness using a digital caliper daily from day 22 after the first CII challenge. Disease severity was assessed using a standardized clinical score based on joint thickness, in a blinded manner, as previously described by us [29, 30]. Briefly, clinical scores were determined as follows: score 0 = normal joint, 1 = paw swelling only, 2 = one joint of one limb along with paw swelling, 3 = multiple joints on a limb involved, and 4 = all joints involved or limb fusion. A clinical score ranging from 0 to 16 was assigned to each mouse by summing the scores of each paw [30, 44].

### Evaluation of serum anti-collagen type II (CII) antibodies

Serum levels of mouse anti-collagen antibodies (autoantibodies) and bovine anti-collagen antibodies (antibodies to the immunizing antigen) were determined by ELISA using a Mouse Anti-mouse Type II Collagen IgG Antibody Assay Kit and Mouse Anti-Bovine Type II Collagen IgG Antibody Assay Kit, respectively, according to the manufacture’s protocol (Chondrex Inc. Redmond, WA, USA). The antibody concentrations in the test samples were calculated by comparison with the optical density (OD) values of standard anti-CII antibody (units/μl).

### Evaluation of serum cytokines and chemokines

Concentrations of a panel of 29 murine cytokines and chemokines were analyzed using the V-PLEX Mouse Cytokine 29-Plex kit on the Meso Scale Discovery (MSD) platform (Meso Scale Diagnostics, Rockville, MD, USA), according to the manufacturer’s protocol. Data was analyzed using Discovery Workbench 4.0 software (Meso Scale Diagnostics). Serial dilutions of specific recombinant cytokines and chemokines were used to establish a standard curve to determine concentrations of each of the analytes measured.

### Evaluation of matrix metalloproteinases (MMP) in the joints

One hind or front joint with clinical symptoms in the CIA mice was collected for protein lysate, and similar joints collected from all other animals in the experiment for relative comparisons. Flash-frozen mouse joints were homogenized using a tissue homogenizer (Omni International, USA) in protein extraction buffer T-PER (Thermo Scientific, USA) containing protease inhibitor cocktail (Cell Signaling Technology, Denver, USA). The homogenates were centrifuged at 10,000×*g*, at 4 °C for 10 min, and supernatants collected, aliquoted, and stored at − 20 °C until use. Total protein amount was estimated in the supernatants using the micro-bicinchoninic acid (BCA) assay (Thermo Scientific, USA) according to the manufacturer’s instructions. The joint tissue lysates were probed in western blots to examine the abundance of MMP-1, MMP-3, MMP-9, and MMP-13, all associated with the pathology of arthritis [45, 46]. The joint tissue lysates (20 μg per sample) were resolved on NuPage 4–12% Bis-Tris protein gels (Invitrogen) and transferred onto nitrocellulose membranes. The membranes were blocked overnight with 5% milk powder (w/v) in TBST (20 mM Tris–HCl pH 75, 150 mM NaCl, 0.1% Tween-20) and probed with antibodies against murine MMP-1, MMP-3, MMP-9, and MMP-13 (all obtained from Abcam). Antibody to GAPDH (Cell Signaling Technologies) was used to determine and normalize for protein loading. Affinity-purified horseradish peroxidase (HRP)-linked secondary antibodies (Cell Signaling, USA) along with Amersham ECL Prime (GE Healthcare) was used for detection. The blots were imaged using Amersham™ Imager 680 blot and gel imager. Densitometry was performed to assess band intensity using Amersham™ Imager 680 analysis software version 2.0. Relative band intensity for each MMP was determined after normalization for protein loading using band intensity for GAPDH, for each sample. GraphPad Prism 7.05 software was used for statistical analyses.

### Histology for cellular infiltration and cartilage degradation

On the day of sacrifice, ankle joints were collected and fixed in 10% buffered formalin for 48 h. One hind joint with clinical symptoms in the CIA mice was collected for histology, and similar joints collected from all other animals in the experiment for relative comparisons. The joints were decalcified using 10% EDTA for 14 days followed by dehydration in increasing concentrations of ethanol. The tissues were embedded in paraffin and serial sagittal sections (5 μm) were obtained. The sections were stained with hematoxylin and eosin (H&E) to determine cellular infiltration in the joints. Safranin-O stain was used to stain proteoglycans, representing the subchondral bone cartilage of the joints. Sections were imaged and processed with a Zeiss imager M2 (Germany) using the Zen 2011 software. The stained sections were scored as previously described [29, 30], in a blinded manner by three independent personnel. A histology score was used to determine the extent of cellular infiltration and integrity of the joints as follows: a score of 0 = normal synovium, 1 = synovial membrane hypertrophy and cell infiltration, 2 = pannus formation and cartilage erosion, 3 = major erosion of the cartilage, and 4 = loss of joint integrity, based on previous studies [29, 30, 47].

### Statistical analysis

GraphPad Prism 7.05 software was used for data analyses. Statistical significance was determined by Kruskal–Wallis one-way analysis of variance (ANOVA) followed by Dunn’s multiple comparison post hoc test. Mann–Whitney *U* test was used for paired analyses to determine the *p* values between any two groups of mice. A *p* value of ≤ 0.05 was considered to be statistically significant.

## Results

### Combination of CUR and VO-enriched diet maximally reduced disease severity and delayed clinical progression in CIA mice

**Fig. 1:**
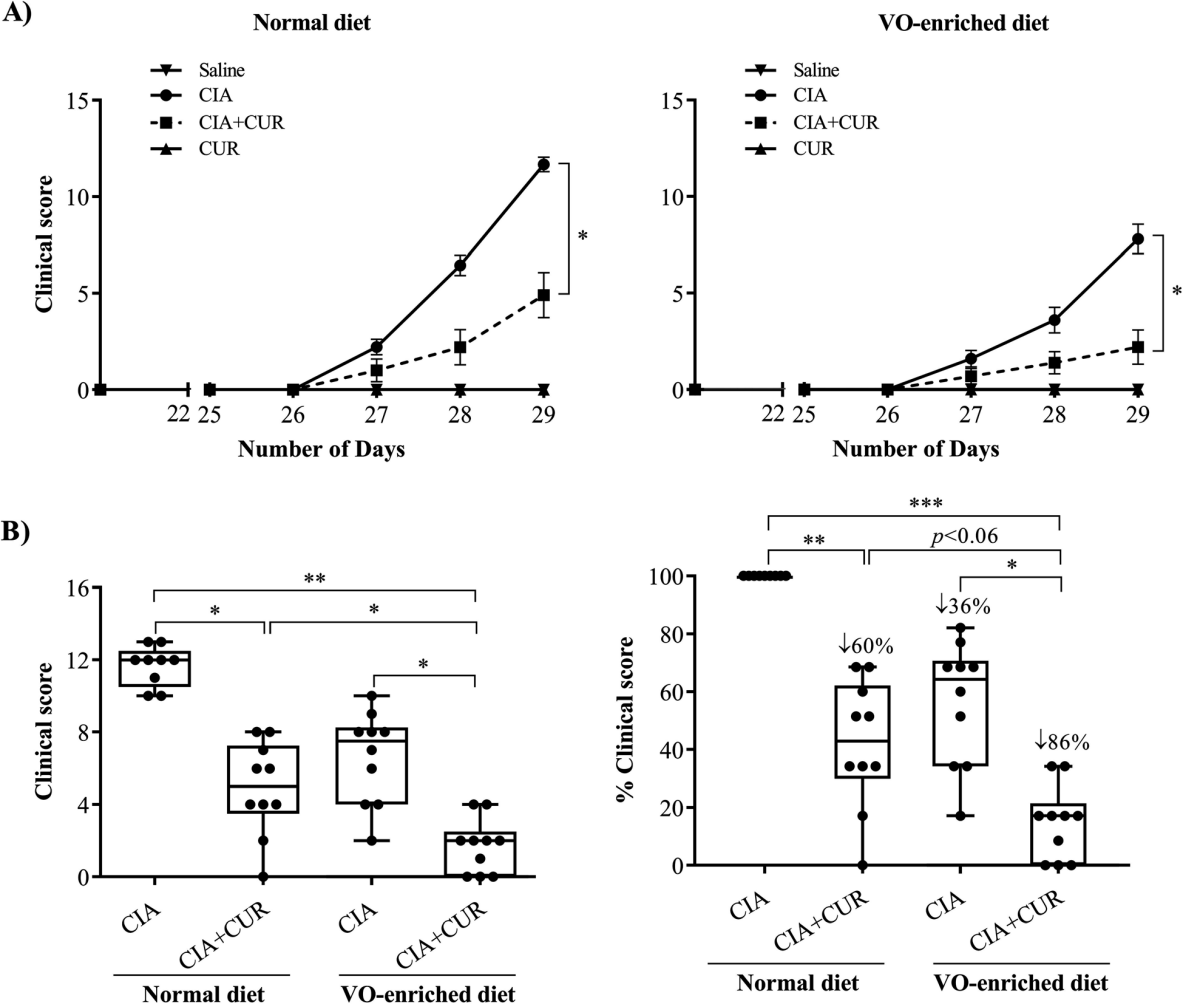
Combination of CUR and VO-enriched diet reduced disease severity and suppressed clinical scores in CIA mice. Saline control and CIA mice under different conditions as indicated were monitored for disease severity and assigned clinical scores from day 21 after the first CII challenge. **a** Line graphs representing the mean clinical scores of mice fed with normal diet and VO-enriched diet, starting from day 1 to day 29. **b** Box plots representing the clinical scores on day 29, and percent clinical scores compared to CIA mice on the normal diet. GraphPad Prism 7.05 software was used for statistical analyses. Kruskal–Wallis one-way ANOVA followed by Dunn’s multiple comparison post hoc test was used to determine the *p* values (*p ≤ 0.05, **p ≤ 0.01, and ***p ≤ 0.001)

Consistent with our previous studies, CIA mice on a normal diet showed typical paw and joint swelling, resulting in significantly higher clinical scores compared to the saline administered control mice (Fig. 1) [29, 30]. The CIA mice on both normal and VO-enriched diet exhibited a gradual increase in clinical scores from day 26 to day 29 after the first CII challenge (Fig. 1a). At the completion of the protocol on day 29, we showed that CIA mice on the VO-enriched diet alone had a ~ 35% reduction of clinical scores compared to mice on a normal diet (Fig. 1b). The administration of CUR reduced the clinical scores by ~ 60% in CIA mice fed a normal diet and > 80% in CIA mice fed the VO-enriched diet (Fig. 1b). CUR administration along with VO-enriched diet significantly decreased the severity of clinical symptoms of CIA, compared to either CUR or VO-enriched diet alone (Fig. 1b). The slope of the line corresponding to mean values of clinical scores from day 26 to day 29 showed that CUR administration in CIA mice fed the VO-enriched diet had the lowest value for the slope of trend line representing disease progression (0.73 ± 0.02), compared to all other groups of CIA mice with values between ~ 1.6 and > 3.9 (Table 1). These results indicated that CUR administration in mice fed the VO-enriched diet had the longest duration of no clinical symptoms, compared to any of the other groups of CIA mice. These results suggested that the combined effect of CUR and VO-enriched diet maximally delayed disease onset.

**Table 1:** Slope of the lines indicating disease progression

Diet type	Experimental groups	Slope of the line
**Normal diet**	CIA	3.92 ± 0.48
	CIA + CUR	1.59 ± 0.28
**VO-enriched diet**	CIA	2.24 ± 0.26
	CIA + CUR	0.73 ± 0.02

### Administration of CUR alone or in combination with a VO-enriched diet suppressed cellular infiltration, synovial hyperplasia, and cartilage degradation in joints of CIA mice

**Fig. 2:**
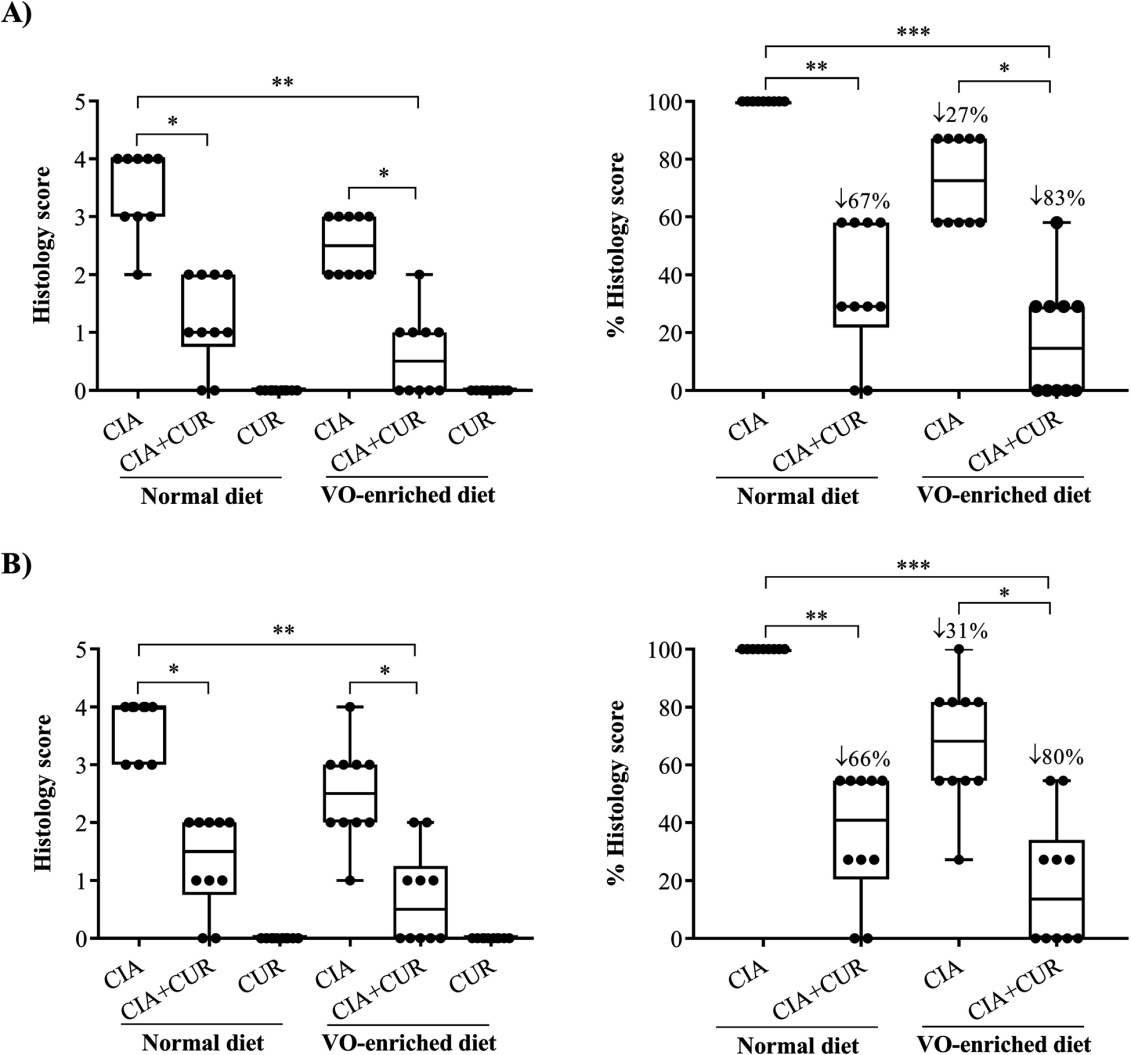
CUR alone, or in combination with a VO-enriched diet, significantly suppressed cellular infiltration and cartilage degradation in joints of CIA mice. Saline control and CIA mice under different conditions as indicated were euthanized by cardiac puncture under anesthesia on day 29 after the first CII challenge. Hind ankle joints were collected and processed for histology. The paraffin-embedded sagittal sections (5 μm) of hind ankle joints were stained with **a** H&E to detect the cellular infiltration and **b** safranin-O for proteoglycan and cartilage degradation. Box plots represent histology scores of H&E-stained and safranin-O-stained joint tissue sections. Percent histology score shown is compared to CIA mice on the normal diet as 100%. GraphPad Prism 7.05 software was used for statistical analyses of histology scores. Kruskal–Wallis one-way ANOVA followed by Dunn’s multiple comparison post hoc test was used to determine the *p* values (*p ≤ 0.05, **p ≤ 0.01, and ***p ≤ 0.001)

Inflammatory arthritis is characterized by an extensive infiltration of inflammatory cells into articular joint/tissues, synovial hyperplasia, and cartilage degradation [29, 30, 45]. We performed histological assessment of cellular infiltration using H&E stain (Fig. 2a) and assessed the integrity of cartilage by safranin-O stain (Fig. 2b), using paraffin-embedded joint sections. As previously described, untreated CIA mice on a normal diet demonstrated extensive cellular infiltration, synovial hyperplasia, loss of joint integrity, and cartilage/proteoglycan degradation, resulting in significantly higher histological scores (3.4 ± 0.3, *p* ≤ 0.01) compared to saline-treated mice (Fig. 2 and Supplementary Fig. 1). CIA mice fed a VO-enriched diet showed ~ 30% reduction in cellular infiltration and cartilage degradation, compared to CIA mice on the normal diet, and the decrease was not statistically significant (Fig. 2). CUR administration significantly reduced the histological scores by ~ 70% in CIA mice fed with the normal diet and by ~ 80% in CIA mice fed with the VO-enriched diet. These results showed that there was no clear difference in the degree of suppression attributable to the VO diet (Fig. 2). Therefore, these results suggested that the suppression of inflammatory cell infiltration, synovial hyperplasia, and cartilage degradation in CIA mice was primarily mediated by the effects of bioavailable CUR.

### Administration of CUR alone or in combination with a VO-enriched diet suppressed the abundance of matrix metalloproteinases in the joints of CIA mice

**Fig. 3:**
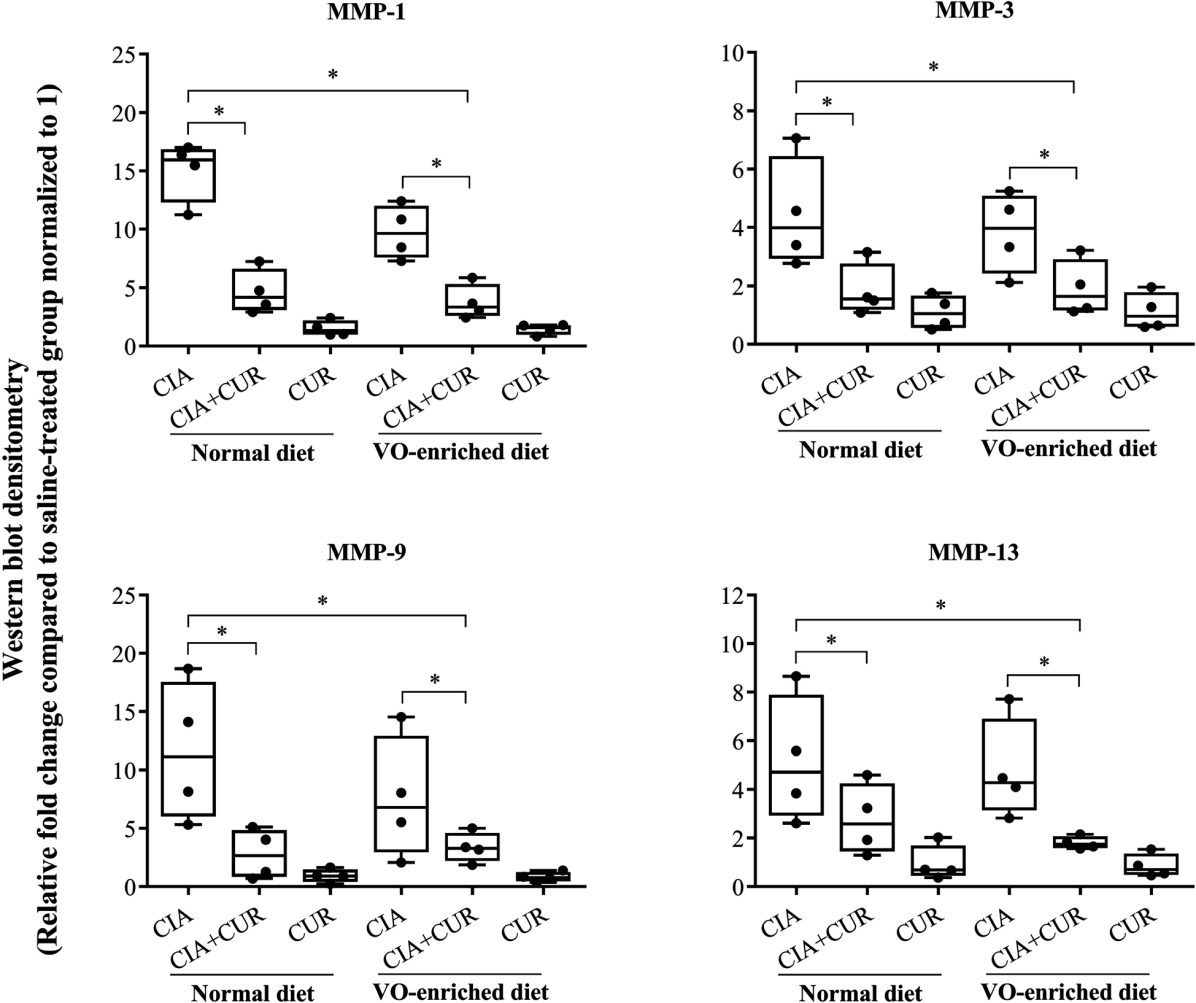
CUR alone, or in combination with a VO-enriched diet, mitigated the abundance of MMPs in the joints of CIA mice. Joint tissue lysates (20 μg protein each) were resolved on NuPage 4–12% Bis-Tris protein gels and probed in immunoblots with antibodies for mouse MMP-1, MMP-3, MMP-9, and MMP-13. Antibody to mouse GAPDH was used to assess protein loading. Densitometry for band intensity was determined using the AmershamTM Imager 680 analysis software version 2.0. Relative band intensity was determined by normalizing to the GAPDH band intensity for each sample. Graphs represent the relative fold change compared to saline-treated mice normalized to 1. GraphPad Prism 7.05 software was used for statistical analyses. Kruskal–Wallis one-way analysis of variance (ANOVA) followed by Dunn’s multiple comparison post hoc test was used to determine the *p* values (*p ≤ 0.05)

MMPs are key mediators of tissue/cartilage degradation in inflammatory arthritis and are known to be elevated in the synovial tissue, fluid, and serum of RA patients [45, 46]. We have previously shown that mRNA expression of specific MMPs, such as MMP-3 and MMP-13, are elevated in the joints of CIA mice [30]. In this study, we evaluated the protein abundance of MMP-1, MMP-3, MMP-9, and MMP-13 in the joint tissue homogenates using western blots. We demonstrated that the protein levels of the MMPs examined were between 4- and 15-fold higher in the joint tissue lysates of CIA mice compared to saline-treated mice, on the normal diet (Fig. 3 and Supplementary Fig. 2). MMP protein abundance decreased modestly and insignificantly in CIA mice fed a VO-enriched diet, compared to those fed a normal diet (Table 2). CUR administration significantly reduced the protein abundance of all MMPs examined by 50–75% in CIA mice, irrespective of their diet (Table 2). These results were consistent with the histological scores with most of the suppression of MMPs in the joints being attributable to CUR administration.

**Table 2:** Percent decrease of MMP abundance in joint tissues

MMPs	% Decrease compared to mean MMP abundance in CIA (normal diet)					
	CIA + CUR		CIA + VO		CIA + VO + CUR	
	% Decrease	***p*** value	% Decrease	***p*** value	% Decrease	***p*** value
**MMP1**	69 ± 4%	*p* < 0.03	40 ± 6.9%	*p* < 0.99	75 ± 3.1%	*p* < 0.01
**MMP3**	58 ± 6.4%	*p* < 0.05	14 ± 9.2%	*p* < 0.99	57 ± 6.8%	*p* < 0.05
**MMP9**	75 ± 5.8%	*p* < 0.04	34 ± 14.3%	*p* < 0.99	71 ± 3.5%	*p* < 0.05
**MMP13**	47 ± 8.9%	*p* < 0.06	10 ± 7.3%	*p* < 0.99	65 ± 1.8%	*p* < 0.01

### Administration of CUR alone or in combination with a VO-enriched diet reduced anti-collagen type II antibodies in the CIA mice

**Fig. 4:**
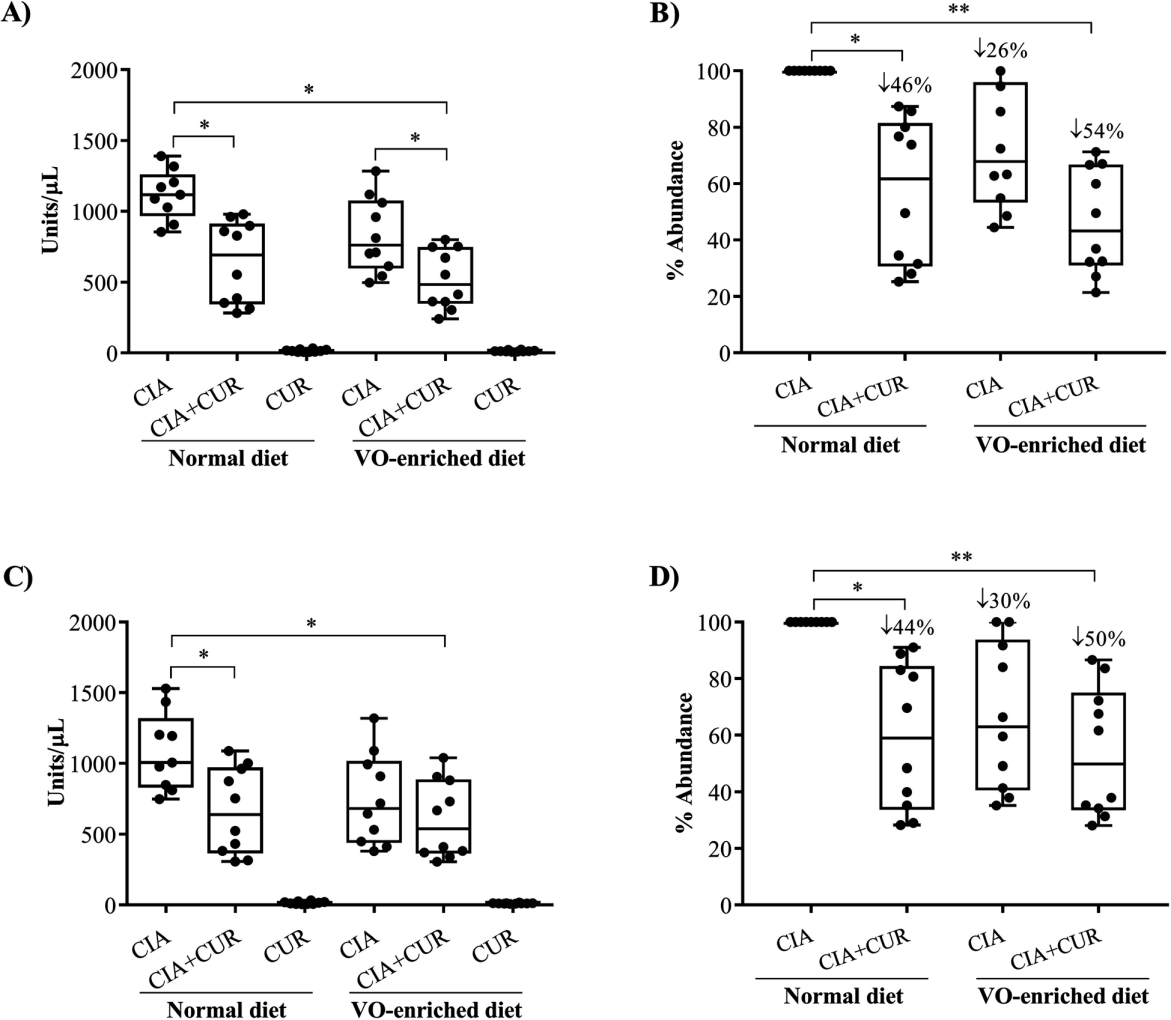
CUR alone, or in combination with a VO-enriched diet, suppressed anti-collagen type II (CII) antibodies in the CIA mice. Saline control and CIA mice under different conditions as indicated were euthanized by cardiac puncture under anesthesia on day 29 after the first CII challenge. Serum concentrations of **a** anti-mouse collagen type II antibodies and **b** anti-bovine collagen type II antibodies were monitored by ELISA. Percent abundance shown is compared to CIA mice on the normal diet. Kruskal–Wallis one-way analysis of variance (ANOVA) followed by Dunn’s multiple comparison post hoc test was used to determine the *p* values and Mann–Whitney *U* test was used to determine the *p* value between any two groups (*p ≤ 0.05 and **p ≤ 0.01)

Immunization of DBA/1J mice with heterologous bovine CII results in the production of antibodies against the bovine CII (immunizing antigen) and murine CII auto-antigen [29, 48]. We therefore measured serum concentrations of both bovine CII and murine CII antibodies by ELISA. Consistent with previous studies [30], serum levels of both anti-mouse CII and anti-bovine CII antibodies were markedly elevated (> 1000 units/μl) in CIA mice compared to saline-treated mice (Fig. 4a and c respectively). Although the VO-enriched diet alone tended to reduce the titers of these antibodies compared to a normal diet, this did not reach statistical significance (Fig. 4b, d). CUR administration significantly reduced the levels of both antibodies by 40–50%, irrespective of diet (Fig. 4b and d respectively). These results were consistent with the histological scores and with MMP protein assessment, with suppression of anti-collagen II antibodies being attributable to CUR administration.

### Serum cytokine profile is modulated distinctly by the administration of CUR alone or in combination with a VO-enriched diet, in CIA mice

**Fig. 5:**
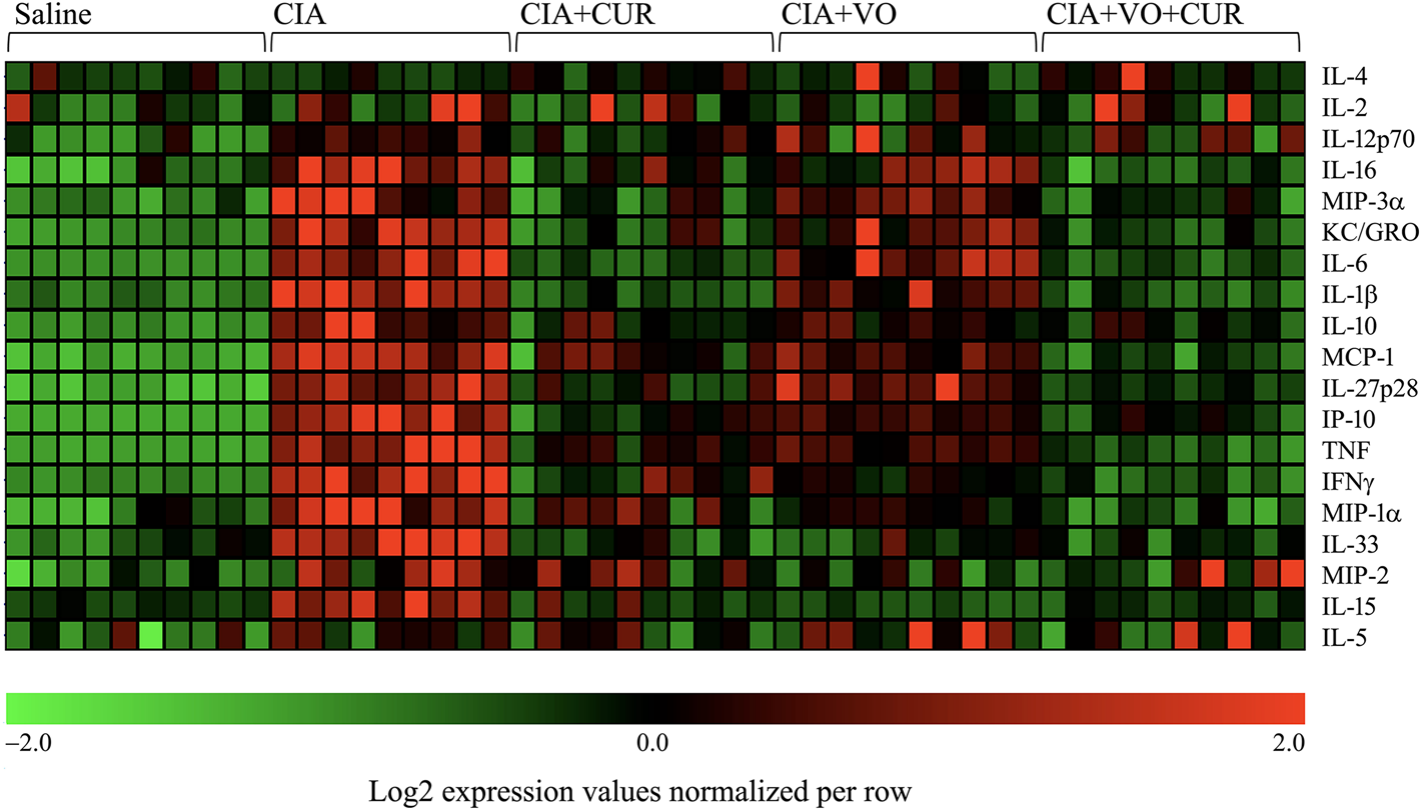
Combination of CUR and VO-enriched diet maximally altered the serum cytokine/chemokine profile in CIA mice. Saline control and CIA mice under different conditions as indicated were euthanized by cardiac puncture under anesthesia on day 29 after the first CII challenge. Concentrations of a panel of 29 cytokines/cytokines were examined in serum, using the V-PLEX mouse multiplex Meso Scale Discovery (MSD) assay kit. Intensity for each analyte was log2 transformed and normalized across each row. The heatmap shows differentially expressed cytokines and chemokines for all samples (*n* = 10 per group), where each column shown represents one mouse (green to red ranges from − 2 to + 2 intensities respectively)

Compared to the normal state, in chronic inflammatory diseases such as RA, there are often changes in the balance between pro- and anti-inflammatory cytokines/chemokines detectable in the serum, although the direction of these changes is not consistent and predictable in all individuals [49]. We have previously defined a panel of cytokine/chemokines that are differentially altered in the serum of CIA mice [29, 30]. In this study, we examined a panel of 29 cytokines and chemokines using the multiplex MSD platform. Our results showed that 19 of the 29 cytokines/chemokines measured were differentially expressed (≥ 2-fold, *p* ≤ 0.05) in the serum of CIA mice, compared to saline-treated control mice (Fig. 5). Consistent with the clinical and histological data, administration of CUR broadly resulted in suppression of multiple inflammatory cytokines/chemokines that were elevated in the CIA mice (Fig. 5, Table 3). The VO-enriched diet alone demonstrated some modulation of the cytokine/chemokine profile towards the non-inflammatory state, but these effects were modest.

**Table 3:** Percent decrease of cytokines and chemokines in serum

Cytokine	% Decrease compared to mean abundance in CIA mice on normal diet					
	CIA + CUR		CIA + VO		CIA + VO + CUR	
	% Decrease	***p*** value	% Decrease	***p*** value	% Decrease	***p*** value
**IFNγ**	58 ± 7.3	*p* < 0.0003	55 ± 4.1	*p* < 0.0003	82 ± 3.3	*p* < 0.0001
**TNF**	45 ± 3.6	*p* < 0.0002	36 ± 2.3	*p* < 0.0002	69 ± 2.8	*p* < 0.0002
**IL-1β**	73 ± 2.6	*p* < 0.001	34 ± 5.3	*p* < 0.0015	75 ± 3.1	*p* < 0.0001
**IL-6**	77 ± 2.7	*p* < 0.002	16 ± 8.4	*p* < 0.211	77 ± 3.0	*p* < 0.002
**IL-33**	76 ± 4.8	*p* < 0.004	70 ± 4.8	*p* < 0.001	61 ± 6.8	*p* < 0.005
**IL-15**	75 ± 6.6	*p* < 0.0003	91 ± 2.5	*p* < 0.097	81 ± 2.9	*p* < 0.0003
**IL-16**	47 ± 6.3	*p* < 0.0005	20 ± 6.2	*p* < 0.0002	60 ± 3.4	*p* < 0.0002
**IL-10**	46 ± 6.3	*p* < 0.041	37 ± 3.7	*p* < 0.002	51 ± 4.4	*p* < 0.0002
**MCP-1**	36 ± 5.9	*p* < 0.0003	22 ± 2.9	*p* < 0.021	55 ± 3.1	*p* < 0.0003
**KC/GRO**	54 ± 8.2	*p* < 0.0002	16 ± 8.0	*p* < 0.112	67 ± 3.8	*p* < 0.0002
**IP-10**	59 ± 4.9	*p* < 0.001	40 ± 1.9	*p* < 0.001	62 ± 3.9	*p* < 0.0001
**MIP-1α**	38 ± 5.8	*p* < 0.001	30 ± 4.8	*p* < 0.031	59 ± 3.4	*p* < 0.0015

**Fig. 6:**
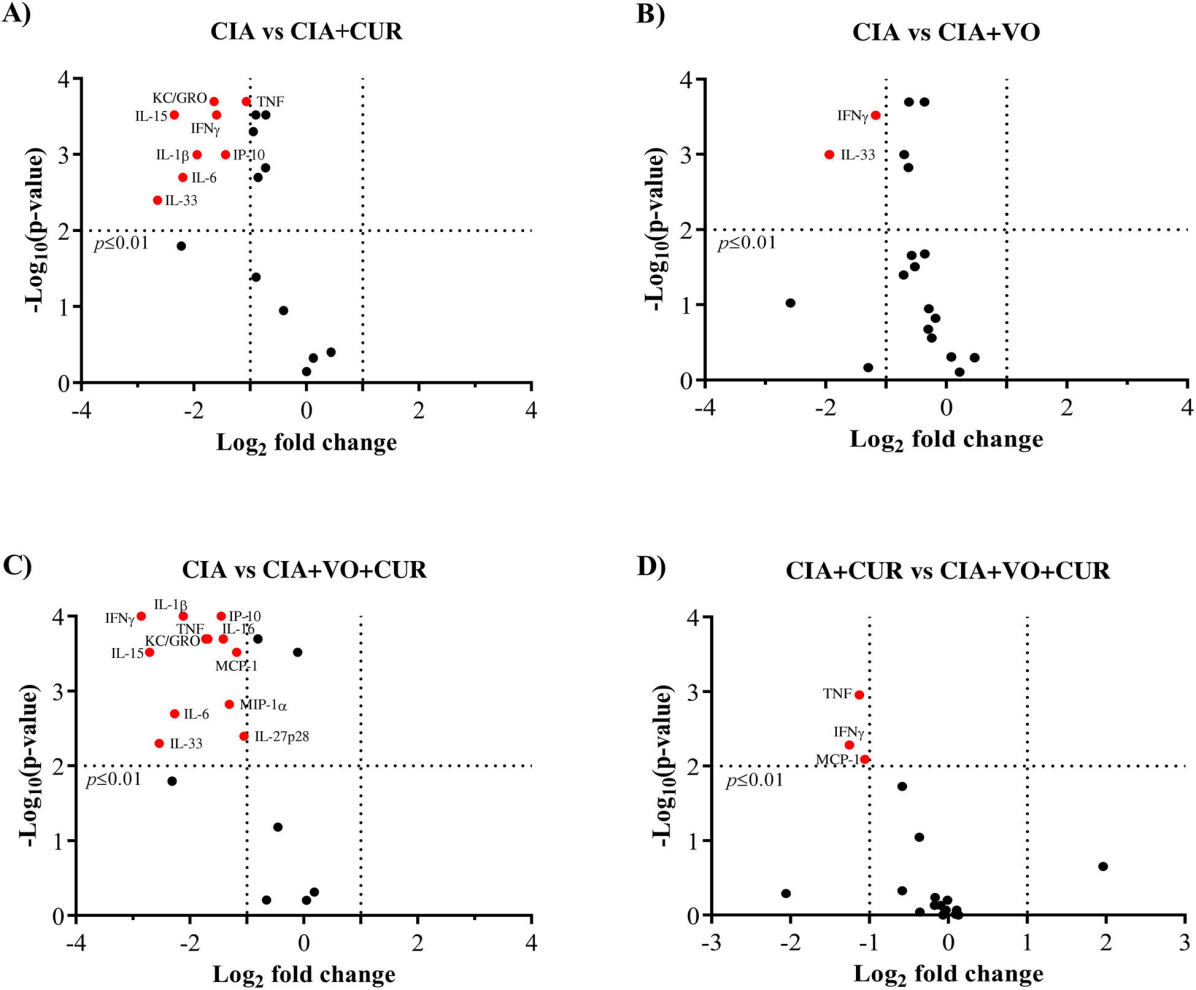
Combination of CUR and VO-enriched enhanced suppression of TNF, IFN-γ, and MCP-1, compared to either CUR or VO-enriched diet alone, in CIA mice. Saline control and CIA mice under different conditions as indicated were euthanized by cardiac puncture under anesthesia on day 29 after the first CII challenge. Concentrations of a panel of 29 cytokines/cytokines were examined in serum, using the V-PLEX mouse multiplex Meso Scale Discovery (MSD) assay kit. Volcano plots shown represent log2 transformed intensity values for each analyte, between two groups of CIA mice **a** on the normal diet, with and without CUR administration, **b** fed normal diet with those on VO-enriched diet, **c** on normal diet with those fed VO-enriched diet and CUR administration, and **d** CUR administration, on normal diet with those fed the VO-enriched diet. Cytokines shown in red are those with > 2-fold change (*p* < 0.01)

**Fig. 7:**
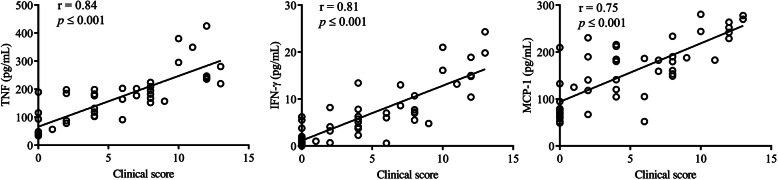
Correlation analysis of clinical scores with serum concentrations of TNF, IFN-γ, and MCP-1. Correlation analysis was performed between clinical scores and the serum concentrations of TNF, IFN-γ, and MCP-1 of each mouse, from saline control and CIA groups under different conditions (CUR alone, VO-enriched diet, and the combination of CUR and VO-enriched diet). Spearman correlation analysis was used to determine the significance of correlation analysis. *p* < 0.05 was considered to be statistically significant

Comparison of the serum cytokine profiles showed that administration of CUR alone suppressed 9 pro-inflammatory cytokines/chemokines by > 2-fold in CIA mice fed the normal diet (*p* < 0.01) (Fig. 6a). These included TNF, IL-1β, IL-6, IL-33, IL-15, IL-16, KC, MIP-α, and IP-10 (Fig. 6a). All of these cytokines/chemokines were reduced between 45 and 77% following CUR administration in the CIA mice on the normal diet (Table 3). In contrast, only IL-33 and IFN-γ levels were significantly suppressed (> 2-fold) in CIA mice fed the VO-enriched diet (Fig. 6b). In contrast, administration of CUR in the CIA mice on the VO-enriched diet significantly reduced the levels of 13 pro-inflammatory cytokines by > 2-fold, between 65 and 85% (Fig. 6c and Table 3). A comparison of cytokine profiles showed that CUR-mediated suppression of serum levels of TNF, IFN-γ, and MCP-1 was significantly enhanced by > 2-fold in CIA mice fed the VO-enriched diet, compared to CIA on the normal diet (Fig. 6d). There was a linear correlation between clinical scores and the serum levels of TNF, IFN-γ, and MCP-1 (Fig. 7).

## Discussion

In this study, we examined the pathophysiological effects of a highly bioavailable form of CUR (Cureit/Acumin™), alone and in combination with a VO-enriched diet, in modulating the onset of CIA. The CIA murine model used is well suited for modeling the preclinical stages of RA, as it not only recapitulates the phenotypic features of human inflammatory arthritis such as joint swelling, inflammation, and destruction of the joint cartilage and bone, but also allows for an exploration of intervention strategies that modulate immune-mediated events that lead to disease development [50–52]. The findings in this study clearly demonstrated that following acclimatization with a VO-enriched diet, bioavailable CUR effectively delayed disease onset and progression in the CIA mice. Although suppression of cellular infiltration and MMPs in the joints, and serum anti-collagen antibodies, was primarily driven by CUR, the combination of CUR and a diet enriched with vitamin D and O3FA showed the maximal benefit in suppressing the clinical symptoms and some of the molecular features of the immune-mediated inflammatory arthritis. In view of the safety and wide availability of these supplements, our results provide compelling rationale for evaluating the effects of the combination of these supplements in individuals at risk for future RA development, such as those who are autoantibody positive but have no evidence of clinically detectable inflammatory arthritis.

The beneficial effects of each of the nutritional supplements used in this study have been demonstrated individually in chronic disease management, particularly for their anti-inflammatory effects [53, 54]. Individually, CUR, vitamin D_3_, and O3FA have been shown to modulate inflammatory responses and control clinical symptoms, both in RA patients and in CIA mouse models [17, 33, 55, 56]. Previous studies have also demonstrated the effects of these nutritional supplements in the control of pain [10–12, 37, 55]. However, despite the extensive literature available for each of the supplements, both from human studies and animal models, there are essentially no studies that have systematically examined the pathophysiological effects of these nutritional supplements when used in combination. Moreover, in current clinical practice, the use of combination DMARD therapy is now well established in the treatment of RA, and patients not uncommonly take combinations of these supplements as complementary therapies.

CUR is a component of the common South-Asian spice turmeric, known to elicit anti-inflammatory effects and used in Ayurvedic medicine [57–60]. CUR by itself has been demonstrated to be beneficial in inflammatory arthritis [35, 61, 62]. A key challenge associated with the medicinal use of CUR is its poor bioavailability [27, 28, 63]. To address this, a highly bioavailable curcuminoid formulation (Cureit/Acumin™) synthesized using Polar-Nonpolar-Sandwich technology with complete natural turmeric matrix by Aurea Biolabs Ltd., (Cochin, Kerala, India) was used in this study [32]. This formulation increases the bioavailability of CUR by 5–10-fold [32, 33]. Cureit/Acumin™, given in well-tolerated doses, was previously shown to reduce disease activity and suppress CRP and RF in a small randomized, placebo-controlled clinical trial in RA patients [33]. Based on this, we used this formulation of CUR in the current study, which focused on examining its modulatory effects in the preclinical “immunological” stage of the CIA model, as it will allow for rapid translation into human studies.

The mechanism of action of CUR-mediated control of inflammation and cartilage degradation has been proposed to be by targeting key signaling pathways such as STAT1, mTOR, and PKCdelta/JNK/c-Jun pathways; control of MMP production; and modulation of the gut-brain axis, in murine models of CIA [10, 26, 35]. CUR modulates innate immune responses by suppressing the expression and production of critical cytokines and chemokines, such as IFN-γ, TNF, IL-1β, IL-6, MCP-1, and IL-8, all of which play a crucial role in the development and pathogenesis of RA, via the NF-κB, STAT, and AP1 signaling cascades in innate immune antigen-presenting cells macrophages and dendritic cells [64, 65]. The immunomodulatory effect of CUR has also been demonstrated in the transition of innate immune-to-adaptive responses, as CUR was shown to suppress the activation, proliferation, and differentiation of naïve CD4^+^ T cells to T helper (Th)1 and Th17 subtypes, and prevent joint and bone destruction [66, 67]. Moreover, CUR promotes the differentiation of naïve CD4^+^ T cells to CD4^+^ CD25^+^ FOXP3^+^ regulatory T cells (Treg), which has been suggested as one of the mechanisms underpinning the ability of CUR to suppress the polarization of immune response to Th1 and Th17 phenotypes [68, 69]. Overall, these studies indicate that CUR modulates both innate and adaptive immune responses associated with the development and progression of RA.

In this study, we showed that bioavailable CUR by itself suppresses the abundance of several MMPs, including MMP-3, in the joints of CIA mice. It is known that MMP-3 promotes the pathology of RA, is enhanced in RA patients [70], and associated with the development of RA [71, 72]. Previous studies have demonstrated that CUR targets the mTOR pathways to intervene in the production of MMP-3 in a CIA rat model [10] and downregulates MMP-3 to inhibit proliferation of synovial cells [73]. Thus, based on the findings of this study, it is likely that bioavailable CUR may prevent the development of RA symptoms in at-risk individuals by suppressing the production of MMP-3.

In the current study, we showed that bioavailable CUR is maximally effective in ameliorating the onset of CIA when combined with vitamin D and O3FA in the diet, with the latter two supplements having only modest effects in the absence of CUR. Vitamin D_3_ and O3FA individually have been shown to mitigate cartilage degradation and inflammation by targeting MMPs, cyclooxygenase (COX)-2 signaling, and p38 MAPK-dependent mechanisms [74–76]. Combined supplementation of D3 and O3FA in the form of cod liver oil has been historically used for treating rheumatic disease [77]. Although attenuation of inflammation with cod liver oil has been established [78], research aimed at delineating the mechanisms underlying the combined effects of D3 and O3FA has largely not been undertaken [10–12, 37, 55]. To our knowledge, this is the first study to evaluate the in vivo biological impact of all three supplements, alone and in combination.

The translation of the findings in the current study to the preclinical stages of human RA faces a number of key challenges. Arguably the biggest of these challenges relates to having the development of clinically detectable/classifiable RA as the primary outcome measure with which to assess the efficacy of specific interventions. Unlike CIA, which has a predictable onset after immunization, the evolution of preclinical RA autoimmunity is unpredictable and can span years in some individuals. In prospective studies, we and others have shown that the development of RA is a rare event requiring prolonged follow-up, even in individuals who are at high risk for developing future disease based on having detectable RA autoantibodies and being family members of RA patients [79, 80]. Thus, there is a critical need to develop quantifiable biomarkers that can reproducibly detect the progression towards disease development, while being responsive enough to reflect the impact of specific interventions. Currently, changes in the levels and antigenic scope of RA autoantibodies, particularly ACPA, are the best available biomarkers with which to follow progression towards disease onset [7, 79–81], but these have proven difficult to recapitulate in murine models. Specific circulating cytokines, particularly TNF and IFN-γ, have been shown to be elevated prior to disease onset, and their level correlated with the accumulation of the RA autoantibodies [80]. Similarly, MCP-1 was demonstrated to be elevated prior to the onset of RA in humans [82], and an antagonist of MCP-1 was shown to prevent the onset of RA on a mouse model [83]. In this study, we showed that the combination CUR and VO-enriched diet maximally suppresses TNF, IFN-γ, and MCP-1 in the CIA model, while substantially reducing the levels on anti-collagen antibodies. Taken together, these findings suggest that TNF, IFN-γ, and MCP-1 may serve as valuable biomarkers in assessing the impact of interventions in reducing the progression towards clinical RA development.

In conclusion, we demonstrate that bioavailable CUR can significantly decrease disease severity and suppress cellular infiltration and MMP abundance, in the joints of CIA mice. However, the combined effect of CUR and VO-enriched diet significantly enhances the control of clinical symptoms and maximally delays the onset and progression of the disease. In addition, the combined effect of CUR and VO-enriched diet significantly suppresses serum levels of critical inflammatory mediators TNF, IFN-γ, and MCP-1, by more than two-fold compared to either CUR or VO-enriched diet alone, in the CIA mice. Thus, the findings of this study provide a rationale for a systematic evaluation of the use of a combination of CUR, vitamin D_3_, and O3FA as a safe and cost-effective intervention with which to reduce the risk of developing inflammatory arthritis in high-risk unaffected individuals who have detectable RA autoantibodies. The findings in this study also provide a framework for developing a biomarker panel with which to evaluate the impact of the intervention.

## Supplementary Information

**Additional file 1: Supplementary Figure 1.** Histology of joint sections. Saline control and CIA mice under different conditions as indicated were euthanized by cardiac puncture under anesthesia on day 29 after the first CII challenge. Joints were deskinned and collected in 10% buffered formalin, decalcified in 10% EDTA and processed for histology. The paraffin embedded sagittal sections (5 μm) of hind ankle joints were stained with H&E to detect the cellular infiltration, and safranin-O for proteoglycan and cartilage degradation. Images shown are representative of sections from each group (*n* = 10). The images were processed using a Zeiss imager M2 using the Zen 2011 software. **Supplementary Figure 2.** Volcano plot analysis comparing CIA mice either fed with VO-enriched diet or CUR alone. Saline control and CIA mice under different conditions as indicated were euthanized by cardiac puncture under anesthesia on day 29 after the first CII challenge. Concentrations of a panel of 29 cytokines/cytokines were examined in serum, using the V-PLEX mouse multiplex Meso Scale Discovery (MSD) assay kit. Volcano plots shown represent log2 transformed intensity values for each analyte. Cytokines shown in red are those with > 2-fold change (*p < 0.01*).

## Data Availability

The datasets during and/or analyzed during the current study available from the corresponding author on reasonable request.
