# Supplementary material for: A bioavailable form of curcumin, in combination with vitamin-D- and omega-3-enriched diet, modifies disease onset and outcomes in a murine model of collagen-induced arthritis
Source: Arthritis Res Ther. 2021 Jan 25;23:39. doi: 10.1186/s13075-021-02423-z (PMC7836561; doi:10.1186/s13075-021-02423-z)

Supplemental Information

Supplementary Figure 1: Histology of joint sections. Saline control and CIA mice under different conditions as indicated were euthanized by cardiac puncture under anesthesia on day 29 after the first CII challenge. Joints were deskinned and collected in 10% buffered formalin, decalcified in 10% EDTA and processed for histology. The paraffin embedded sagittal sections (5 µm) of hind ankle joints were stained with H&E to detect the cellular infiltration, and safranin-O for proteoglycan and cartilage degradation. Images shown are representative of sections from each group (n = 10). The images were processed using a Zeiss imager M2 using the Zen 2011 software.

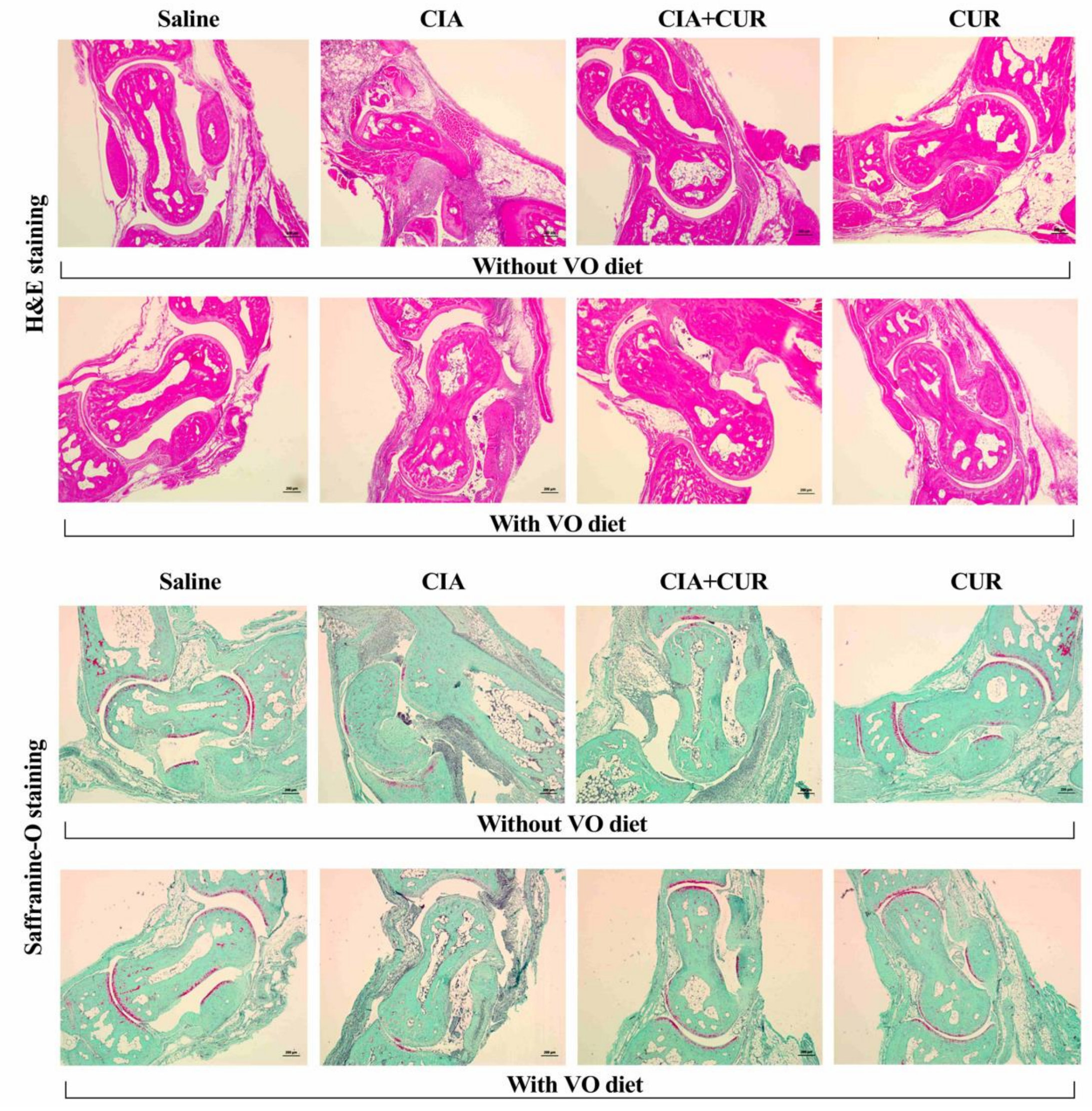

Supplementary Figure 2: Volcano plot analysis comparing CIA mice either fed with VO-enriched diet or CUR alone. Saline control and CIA mice under different conditions as indicated were euthanized by cardiac puncture under anesthesia on day 29 after the first CII challenge. Concentrations of a panel of 29 cytokines/cytokines were examined in serum, using the V-PLEX mouse multiplex Meso Scale Discovery (MSD) assay kit. Volcano plots shown represent log2 transformed intensity values for each analyte. Cytokines shown in red are those with >2-fold change (*p<0.01*).

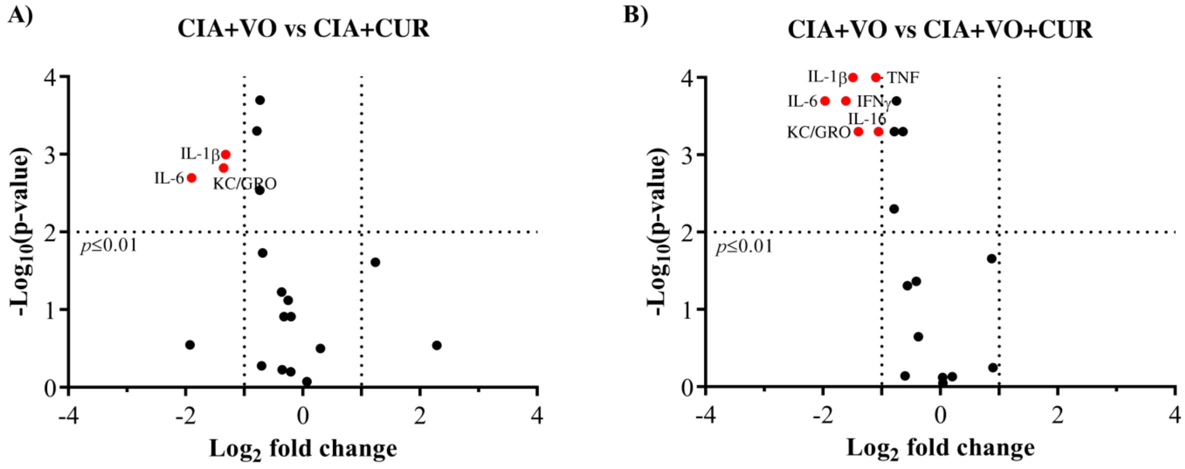

Supplement: Supplementary file 1 — Additional file 1: Supplementary Figure 1. Histology of joint sections. Saline control and CIA mice under different conditions as indicated were euthanized by cardiac puncture under anesthesia on day 29 after the first CII challenge. Joints were deskinned and collected in 10% buffered formalin, decalcified in 10% EDTA and processed for histology. The paraffin embedded sagittal sections (5 μm) of hind ankle joints were stained with H&E to detect the cellular infiltration, and safranin-O for proteoglycan and cartilage degradation. Images shown are representative of sections from each group (n = 10). The images were processed using a Zeiss imager M2 using the Zen 2011 software. Supplementary Figure 2. Volcano plot analysis comparing CIA mice either fed with VO-enriched diet or CUR alone. Saline control and CIA mice under different conditions as indicated were euthanized by cardiac puncture under anesthesia on day 29 after the first CII challenge. Concentrations of a panel of 29 cytokines/cytokines were examined in serum, using the V-PLEX mouse multiplex Meso Scale Discovery (MSD) assay kit. Volcano plots shown represent log2 transformed intensity values for each analyte. Cytokines shown in red are those with > 2-fold change (p < 0.01). [file 13075_2021_2423_MOESM1_ESM.pdf]
